# Nonsurgical Management of an Extensive Perforative Internal Root Resorption with Calcium-Enriched Mixture Cement

**Published:** 2014-12-24

**Authors:** Ehsan Esnaashari, Arezou Pezeshkfar, Mahta Fazlyab

**Affiliations:** a * Department of Endodontics, Dental Branch, Islamic Azad University, Tehran, Iran; *; b * Department of Periodontology, International Campus, School of Dentistry, Tehran University of Medical Sciences, Tehran, Iran; *; c *Iranian Center for Endodontic Research, Research Institute of Dental sciences, Shahid Beheshti University of Medical Sciences, Tehran, Iran*

**Keywords:** Calcium-Enriched Mixture Cement, CBCT, CEM Cement, Cone-Beam Computed Tomography, Internal Root Resorption, Root Resorption

## Abstract

Internal inflammatory root resorption (IIRR) is a rare condition of the root canal and if it is left untreated it may lead to destruction of the surrounding dental hard tissues. Odontoclasts are responsible for this situation which can potentially perforate the root. Many initiating factors have been mentioned for IIRR, almost all causing chronic inflammation in the vital pulp. IIRR is usually symptom free, but in cases of root perforation, a sinus tract usually forms. The prognosis of treatment depends on the size of lesion with small lesions being managed with good prognosis. However, in case of notable destruction of the tooth, the prognosis is poor and tooth extraction may become inevitable. This report represents the management of an extensive perforative IIRR that was successfully sealed with calcium-enriched mixture (CEM) cement. After 12 months the tooth was still symptomless and in function.

## Introduction

Internal inflammatory root resorption (IIRR) is a well-known disease that ultimately perforates the root and destroys the surrounding dental hard tissues [1]. However, its etiology is only partially understood. Internal and cervical invasive resorptions are often incorrectly confused [1, 2].

Unlike root resorptions initiating from the surrounding root tissues, IIRR tends to be self-limiting after the whole root canal pulp tissue becomes necrotic due to advancing root canal infection [2]. When correctly diagnosed, the IIRR is relatively simply treated with good prognosis [3, 4]. However, in cases where the resorption has perforated the root, the tooth structure may have become too weak, and elimination of infection can also be more difficult [3].

From etiologic point of view, under normal conditions predentin layer and odontoblast cells covering the mineralized dentine, act as a barrier that separates pulpal cells with resorbing potential (*aka. *dentinoclasts) from the dentin inside the root canal [5-7]. However, trauma or other events such as pulpitis, pulpotomy, cracked tooth, tooth transplantation, restorative procedures, invagination, orthodontic treatment and even a Herpes zoster viral infection [1, 6, 7], may lead to separation of this protective layer from the dentin surface and eventually lead to differentiation of dentinoclasts and dentin resorption [5, 8]. Damage to the cells of the odontoblastic layer may occur because of inflammation as a reaction of the pulp connective tissue to infection approaching through dentin (caries) [2]. The fact that IIRR is also found in the middle and apical parts of the roots of mandibular premolars and molars which are well protected against trauma, may confirm that pulpal inflammation can initiate this phenomena [9].

The clinical characteristics of internal root resorption depend on the degree and stage of the resorption. Although most teeth with IIRR are symptom-free, during active progression of the resorption the tooth is at least partially vital and may show typical symptoms of pulpitis [6]. If the resorption occurs in or near the crown, it may show as a pinkish or reddish color through the crown indicating that only a thin layer of enamel is left over the highly vascularized connective tissue composed of resorbing cells [1, 6, 9]. Untreated teeth often turn gray/dark gray if the pulp becomes necrotic [9]. Perforation of the root is usually followed by the development of a sinus tract [6, 7]. Radiographically, a radiolucent, round and symmetrical widening of the root canal space through which the original canal shape can no longer be observed, is a sign of IIRR [1, 8]. The advent of cone-beam computed tomography (CBCT) has enhanced radiographic diagnosis. Several case reports and case series have proved the usefulness of CBCT in diagnosing and managing IIRR [10, 11].

**Figure 1:**
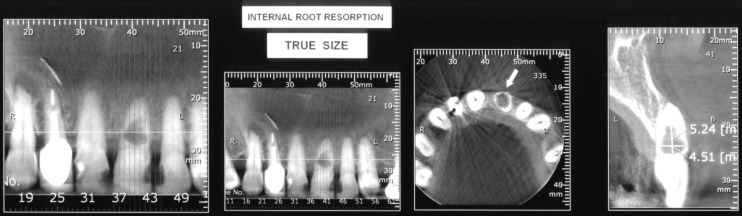
CBCT image showing the extent of internal inflammatory root resorption in the maxillary left central incisor

Instrumentation and cleaning of the root canal space of teeth with IIRR is a challenge different from those of normal endodontic treatment. In case of actively resorbing vital tissue, excessive bleeding makes it difficult to locate the root canal openings [1]. However, irrigation with sodium hypochlorite (NaOCl) or inter appointment calcium hydroxide (CH) dressing (to control bleeding, and to necrotize residual pulp tissue and to make the necrotic tissue more soluble to NaOCl [6, 9]) will in most cases help to reduce the bleeding [6, 7].

After a short-term application of CH, treatment of the perforated IIRR is followed by filling the resorption cavity with mineral trioxide aggregate (MTA) because of its antimicrobial properties, profound seal and being very well tolerated by the tissues [6].

Calcium-enriched mixture (CEM) cement is a tooth-colored cement with clinical applications similar to MTA. It offers proper sealing ability, antimicrobial properties, hard tissue induction properties and shorter setting time, greater flowability and lower film thickness than MTA [12].

The present report reviews the diagnosis (using CBCT), and nonsurgical treatment (using CEM cement) of a perforative IIRR that had developed a sinus tract and represents the one-year outcome of treatment.

## Case Report

An elderly lady in her seventies asked for a dental visit, with her chief complain being dull pain and swelling in the upper-lip area. On a regular assessment she was healthy and used to take 1 mg cortisol daily for her rheumatoid arthritis. Intra oral examination revealed a normal oral hygiene. The upper lip was swelled and dense on palpation. The vestibule was filled with swelling and a sinus tract was evident in periradicular area of the maxillary left central incisor. The orthograde periapical radiography showed a large lucency within the limitations of the canal. Also CBCT was indicated for evaluation of the extents of the intracanal lesion and also the periradicular lucency (Figure 1), the latter suggesting the communication of the inner pulpal space with the outer periradicular area through a perforation zone. The tooth was not responsive to pulp vitality tests [cold testing with ENDO-ICE frozen gas (Coltene/Whaledent Inc. Mahwah, NJ, USA) and heat testing with a hot burnisher]. Percussion did not elicit any response while the periradicular area was tender upon palpation. Considering the wide lucency within the tooth canal and presence of a sinus tract that could be traced up to the mid-root area and a large periradicular area, the diagnosis was IIRR that had perforated the canal wall and ended up in pulp necrosis.

**Figure 2:**
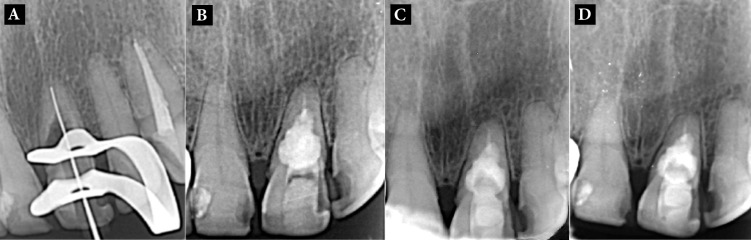
*A)* Endodontic treatment and working length determination; *B)* Post-operative radiography, the root canal is filled with CEM cement; *C)* Six-month recall; *D)* One-year recall; the tooth is absolutely functional and symptom-free

The treatment plan including cleaning of the canal space and its filling with CEM cement was explained for the patient and an informed consent was taken. At the treatment session the tooth was anesthetized with buccal infiltration of 2% lidocaine containing 1:80000 epinephrine (Darupakhsh, Tehran, Iran). After mouth rinse with 0.2% chlorhexidine gluconate (Shahrdaru, Tehran, Iran), a classic access cavity was prepared. The canal was negotiated with a # 15 K-file (Dentsply Maillefer, Ballaigues, Switzerland) and an excessive bleeding was observed. After determination of an estimated working length (WL) (Figure 2A), the canal space was filled with 5.25% NaOCl and was prepared with RaCe rotary instruments (FKG Dentaire, La-Chaux-de Fonds, Switzerland) up to 50/0.04 as the master apical file. To dissolve the hyperemic resorptive tissue in the non-accessible areas, CH powder (Golchay, Tehran, Iran) was mixed with normal saline to prepare a creamy paste that was transferred to the canal with a lentulo spiral (Mani Inc., Shioya-gun, Japan). The tooth was temporarily restored and two weeks later along with healing of the sinus tract, the patient’s symptoms faded away. During the second appointment the canal was rinsed with saline and dried. Then, the whole canal space was filled with CEM cement (BioniqueDent, Tehran, Iran) mixed according to user’s instruction. Confirmation radiography was taken (Figure 2B) and after permanent crown restoration the patient was put on a scheduled follow-up. After six (Figure 2C) and 12 months (Figure 2D), the patient was symptom-free and the tooth was in function.

## Discussion

The present report revealed the successful nonsurgical management of a perforative IIRR lesion in upper left central incisor using CEM cement. After one year, the tooth was symptomless and functional.

IIRR is characterized by the resorption of the internal aspect of the root by multinucleated giant cells (dentinoclasts/odontoclasts) [9]. IIRR is divided into a transient type and a progressive type, with the latter requiring continuous stimulation by infection [7]. Different theories are mentioned regarding the pulpal granulation tissue involved in internal resorption. The most logical explanation is that pulp tissue becomes inflamed due to an infected coronal pulp space [6]. IIRR takes place only if the odontoblastic layer and predentin are lost or altered [13]. Trauma may be an initiating factor. Another reason for the loss of the protective predentin layer might be the extreme heat produced when cutting dentin during restorative cavity preparation without an adequate water spray [2, 9].

Once the diagnosis of IIRR is made and the tooth is deemed restorable with a reasonable prognosis, conventional root canal treatment is the treatment of choice [6, 14]. While preparation of the access cavity and the root canal should be conservative to avoid further weakening of the already compromised tooth, obturation of the affected tooth is a notable clinical challenge. Moreover, the shape of the resorption defect usually renders it inaccessible to direct mechanical instrumentation [6, 7]. Another issue is that in vital teeth bleeding from the inflamed granulation tissues might impair visibility during the initial stages of chemomechanical debridement [14]. CH is a very good candidate for this purpose as it is antibacterial and effective against treatment-persistent bacteria; moreover it acts synergistically in conjunction with NaOCl to remove organic debris from the root canal [14, 15]. In the present case two-week intracanal medication with CH ensured disinfection of the necrotic root canal and gave space for healing of the sinus tract.

In the next stage, treatment procedure is followed by obturation of the canal with an appropriate root-filling material to prevent it from reinfection. IIRR defects can be difficult to obturate due to intra canal irregularity [4]. Thus the obturation material should be flowable especially in situations when the root wall has been perforated [16]. MTA is considered as the material of choice because of its biocompatibility and effectiveness in repairing furcation and lateral root perforations [17, 18]. Above all it supports almost complete regeneration of the periodontium [17, 19]. However, MTA has some drawbacks including potential tooth discoloration that may not be suitable for using in anterior teeth [20].

CEM is an endodontic filling material with its major components being calcium oxide (CaO), sulfur trioxide (SO_3_), phosphorous pentoxide (P_2_O_5_), and silicon dioxide (SiO_2_) [20]. The flow, film thickness, and primary setting time of this biomaterial are favorable [21]. The sealing ability of CEM is similar to MTA [22]. It has the ability to promote hydroxyapatite formation in saline solution and might promote the process of differentiation in stem cells and induce cementogenesis [23]. Moreover CEM cement causes almost no discoloration compared to MTA [24]. The follow-up results of the current case proved all these facts.

Also using CBCT for diagnosis and determining the extent of the IIRR lesion in this case cannot be overlooked. The advent of CBCT has improved the clinicians’ diagnostic capability for IIRR [10]. Nevertheless, IIRR is often asymptomatic, and painful symptoms do not appear until an advanced stage of the lesion [9]. Thus, the clinician’s ability to detect this pathologic entity must rely heavily on the use of radiographs in routine oral examinations [6, 7]. Although the advent of CBCT provides an important adjunctive diagnostic tool for differentiating between IIRR and external cervical resorption, it does not break new ground from a treatment perspective.

## Conclusion

Internal inflammatory root resorption can be successfully treated provided it is diagnosed in time and treated using proper strategy and biomaterials. CEM cement is a good candidate for sealing and filling the internally resorbed teeth.

## References

[1] Fuss Z, Tsesis I, Lin S (2003). Root resorption--diagnosis, classification and treatment choices based on stimulation factors. Dent Traumatol.

[2] Gabor C, Tam E, Shen Y, Haapasalo M (2012). Prevalence of internal inflammatory root resorption. J Endod.

[3] Trope M (2000). Luxation injuries and external root resorption--etiology, treatment, and prognosis. J Calif Dent Assoc.

[4] Mohammadi Z, Yazdizadeh M, Shalavi S (2012). Non-Surgical Repair of Internal Resorption with MTA: A Case Report. Iran Endod J.

[5] Consolaro A (2013). The four mechanisms of dental resorption initiation. Dental Press J Orthod.

[6] Patel S, Ricucci D, Durak C, Tay F (2010). Internal root resorption: a review. J Endod.

[7] Trope M (2002). Root resorption due to dental trauma. Endodontic topics.

[8] Al-Momani Z, Nixon PJ (2013). Internal and external root resorption: aetiology, diagnosis and treatment options. Dent Update.

[9] Haapasalo M, Endal U (2006). Internal inflammatory root resorption: the unknown resorption of the tooth. Endodontic topics.

[10] Kamburoglu K, Kursun S (2010). A comparison of the diagnostic accuracy of CBCT images of different voxel resolutions used to detect simulated small internal resorption cavities. Int Endod J.

[11] Bhuva B, Barnes JJ, Patel S (2011). The use of limited cone beam computed tomography in the diagnosis and management of a case of perforating internal root resorption. Int Endod J.

[12] Asgary S, Kamrani F (2008). Antibacterial effects of five different root canal sealing materials. Journal of Oral Science.

[13] Brito-Junior M, Quintino AF, Camilo CC, Normanha JA, Faria-e-Silva AL (2010). Nonsurgical endodontic management using MTA for perforative defect of internal root resorption: report of a long term follow-up. Oral Surg Oral Med Oral Pathol Oral Radiol Endod.

[14] Siqueira JF Jr, Guimaraes-Pinto T, Rocas IN (2007). Effects of chemomechanical preparation with 25% sodium hypochlorite and intracanal medication with calcium hydroxide on cultivable bacteria in infected root canals. J Endod.

[15] McGurkin-Smith R, Trope M, Caplan D, Sigurdsson A (2005). Reduction of intracanal bacteria using GT rotary instrumentation, 525% NaOCl, EDTA, and Ca(OH)2. J Endod.

[16] Gencoglu N, Yildirim T, Garip Y, Karagenc B, Yilmaz H (2008). Effectiveness of different gutta-percha techniques when filling experimental internal resorptive cavities. Int Endod J.

[17] Torabinejad M, Hong CU, Pitt Ford TR, Kaiyawasam SP (1995). Tissue reaction to implanted super-EBA and mineral trioxide aggregate in the mandible of guinea pigs: a preliminary report. J Endod.

[18] Asgary S, Motazedian HR, Parirokh M, Eghbal MJ, Kheirieh S (2013). Twenty years of research on mineral trioxide aggregate: a scientometric report. Iran Endod J.

[19] Katsamakis S, Slot DE, Van der Sluis LW, Van der Weijden F (2013). Histological responses of the periodontium to MTA: a systematic review. J Clin Periodontol.

[20] Parirokh M, Torabinejad M (2010). Mineral trioxide aggregate: a comprehensive literature review--Part III: Clinical applications, drawbacks, and mechanism of action. J Endod.

[21] Asgary S, Eghbal MJ, Parirokh M, Ghoddusi J, Kheirieh S, Brink F (2009). Comparison of mineral trioxide aggregate's composition with Portland cements and a new endodontic cement. J Endod.

[22] Asgary S, Ahmadyar M (2013). Vital pulp therapy using calcium-enriched mixture: An evidence-based review. J Conserv Dent.

[23] Asgary S, Eghbal MJ, Parirokh M, Ghoddusi J (2009). Effect of two storage solutions on surface topography of two root-end fillings. Aust Endod J.

[24] Nosrat A, Asgary S, Eghbal MJ, Ghoddusi J, Bayat-Movahed S (2011). Calcium-enriched mixture cement as artificial apical barrier: A case series. Journal of Conservative Dentistry.

